# Assessing HVAC airflow modulation strategies to reduce short-term aerosol transmission in office environments

**DOI:** 10.1038/s41598-025-08394-4

**Published:** 2025-07-04

**Authors:** Mina A. Saad, Amr Hassan, Ahmed Hanafy, Mahmoud Salem, Micheal William

**Affiliations:** 1 Mechanical Engineering Department, College of Engineering and Technology, Arab Academy for Science, Technology and Maritime Transport, Alexandria, 60274446 Egypt; 2 Marine Engineering Department, College of Engineering and Technology, Arab Academy for Science, Technology and Maritime Transport, Alexandria, 60274446 Egypt; 3 Mechanical Engineering Department, College of Engineering and Technology, Arab Academy for Science, Technology and Maritime Transport, Smart Village, Giza, 60274446 Egypt

**Keywords:** Airborne transmission, CFD, Discrete phase model, Ventilation optimization, Social distancing, Aerosol dispersion, Computational models, Viral infection

## Abstract

Airborne transmission of respiratory pathogens in indoor environments remains a significant global health challenge. While existing research broadly addresses ventilation effectiveness, there is a critical need to understand how specific diffuser placements influence early-phase aerosol dispersion immediately following a cough event. This study uses Computational Fluid Dynamics (CFD) with an Eulerian–Lagrangian approach and the Discrete Phase Model to analyze initial droplet transport, evaporation, and nuclei concentration under different air distribution configurations. The results demonstrate that conventional parallel exhaust configurations, though effective at reducing overall particle mass, can fail to control the lateral spread of infectious nuclei in the short term. In contrast, placing exhaust diffusers above the cough source reduces the lateral particle spread by approximately 40% compared to conventional layouts. Additionally, maintaining the WHO-recommended two-meter distance results in an 82–89% reduction in particle number concentration during the early dispersion phase. These findings underscore the importance of diffuser placement for controlling short-term particle dispersion immediately after a cough event in mechanically ventilated office environments. The study’s scope is limited to early-phase dispersion dynamics within a 10-second simulation period, and further research is needed to assess long-term aerosol suspension, removal mechanisms, and infection risk. Nonetheless, the results offer practical insights for HVAC design and support the integration of ventilation strategies with physical distancing measures to reduce near-field exposure risks.

## Introduction

Respiratory diseases, propagated by airborne pathogens, present a persistent global health threat. The recent COVID-19 pandemic, driven by SARS-CoV-2, starkly emphasized the urgent need for effective viral transmission control within indoor environments. Originating in Wuhan, China, in late 2019, COVID-19 rapidly escalated into a worldwide crisis, leading to widespread illness and mortality and spurring extensive research into transmission dynamics and preventative measures^1,2^. SARS-CoV-2, like other respiratory pathogens, spreads through airborne particles released when an infected individual coughs, sneezes, talks, or breathes. While larger droplets settle quickly, smaller aerosolized particles can remain suspended for extended periods, traveling significant distances and increasing infection risk^3,4^. Consequently, understanding how indoor air systems influence airborne pathogen concentrations and transmission rates is critical for mitigating their spread^5^. While extensive research has examined ventilation effectiveness in healthcare and high-occupancy settings^6,7^, comparatively fewer studies have explored the influence of HVAC diffuser placement on early-phase aerosol dispersion in mechanically ventilated office environments. Prior research often focuses on overall ventilation efficiency or generalized air mixing, but detailed analyses of how supply and exhaust diffuser arrangements affect the short-term spread of cough-generated particles remain limited. This study aims to address this knowledge gap by investigating the role of airflow modulation strategies in influencing early aerosol transport, providing insights relevant to HVAC design and short-term exposure risk reduction.

Despite the well-established role of ventilation in mitigating airborne transmission, a significant knowledge gap persists in understanding how office-specific airflow dynamics influence nuclei-driven transmission. Prior studies have predominantly focused on healthcare or high-occupancy settings^5,8^, often neglecting the complex interplay between droplet nuclei behavior and realistic HVAC configurations in typical workspaces. This oversight limits our ability to optimize air distribution specifically for office environments, where prolonged exposure in a confined space presents a unique transmission challenge.

This study directly addresses this critical gap by employing Computational Fluid Dynamics (CFD), a powerful tool for simulating airflow patterns and aerosol dispersion^9,10^. By coupling Eulerian–Lagrangian modeling with nuclei-specific concentration analysis, our research offers a comprehensive framework to optimize office air distribution. This engineering analysis provides crucial insights into public health interventions by informing actionable strategies for indoor infection control. Specifically, this multidisciplinary approach integrates principles from fluid engineering (CFD), building environmental design (HVAC optimization), and occupational health (aerosol exposure evaluation), with the ultimate goal of translating these engineering insights into practical recommendations for minimizing airborne transmission risks in office settings, aligning with broader public health guidelines^11^.

## Literature review

### Airborne transmission of respiratory pathogens

The transmission of respiratory diseases, such as COVID-19, is primarily driven by the expulsion of respiratory droplets and aerosols from infected individuals. These droplets can carry infectious agents, including viruses like SARS-CoV-2, and spread through both short- and long-range pathways^12,13^. The size distribution of these droplets plays a critical role in determining their behavior, with larger droplets settling quickly due to gravity, while smaller aerosols can remain airborne for extended periods, increasing the risk of inhalation by others^3,4^. The role of air distribution systems in controlling the spread of airborne pathogens has been studied. Nissen et al.^6^ found evidence of SARS-CoV-2 in the ventilation ducts of COVID-19 hospital wards, confirming the virus’s potential for airborne transmission. Similarly, Zhang et al.^5^ demonstrated that airflow is a primary mechanism for SARS-CoV-2 transmission, emphasizing the importance of proper air distribution and the use of surgical masks in public spaces. These findings underscore the need for effective strategies to reduce the concentration of airborne pathogens in indoor environments.

### CFD modeling of airborne transmission

Computational Fluid Dynamics (CFD) has emerged as a crucial tool for understanding how respiratory droplets and aerosols disperse in indoor environments. However, existing CFD models often oversimplify droplet physics. For instance, they may neglect nuclei behavior or assume static cough parameters. For instance, D’Alessandro et al.^14^ found that only 11% of studies validate evaporation dynamics, leading to inaccurate lifetime predictions. One study improves upon previous work by integrating the Rosin–Rammler distribution with stochastic tracking to accurately capture nuclei dispersion, addressing a recognized gap in recent research^15^.

CFD simulations enable researchers to model intricate airflow patterns, predict the movement of airborne particles, and assess how effectively ventilation systems reduce transmission risks^9,10^. Zhao et al.^16^ used CFD to simulate the spread of COVID-19 aerosols in a university café, demonstrating how ventilation systems can influence the dispersion of infectious particles. Their findings highlighted the limitations of existing ventilation systems in healthcare settings, particularly in controlling the spread of aerosols generated by coughing.

Further studies have explored the impact of ventilation configurations on airborne transmission in various indoor settings. Mirzaie et al.^17^ used CFD to analyze COVID-19 spread in classrooms, with and without partitions. They discovered that higher ventilation rates reduced droplet trapping time and that partitions could partially prevent infection. Similarly, Motamedi et al.^18^ developed a framework for assessing infection probabilities in confined spaces, demonstrating that ventilation configurations significantly influence pathogen dispersion and that single-ventilation (SV) and no-ventilation (NV) scenarios present heightened infection risks. Ugarte-Anero et al.^19^ conducted a numerical investigation using Computational Fluid Dynamics (CFD) to model exhaled aerosol dispersion in a classroom, specifically comparing no, natural, and mechanical ventilation schemes. Their multi-phase study, based on Eulerian–Lagrangian techniques with the k-Shear Stress Transport (SST) turbulence model, simulated teacher speech (4 m/s) and student sneeze (70 m/s max) events. Results demonstrated mechanical ventilation’s superior efficacy in controlling aerosol presence and reducing deposition, evidenced by lower particle accumulation and slower evaporation compared to natural ventilation, while also being significantly more energy-efficient.

### Ventilation strategies and social distancing

Reducing airborne transmission has been a key focus of recent research. Biswas et al. investigated the risk of COVID-19 transmission in elevators under various scenarios, discovering that an exhaust fan significantly lowered the risk of infection in hot, dry conditions. Their study underscored the importance of evaporation dynamics and forced circulation ventilation in accelerating droplet evaporation and reducing viral concentrations.

Recent critiques argue that the 2-m rule inadequately addresses nuclei-driven transmission, as shown by Issakhov et al.^20^ where particles traveled > 5 m in turbulent airflow. While partitions reduce droplet spread^17^, their impact on nuclei remains untested. This study resolves this by quantifying nuclei concentration at incremental distances (Fig. 13), demonstrating that 2-m distancing reduces exposure by 82–89% only when combined with optimized exhaust placement—a finding absent in prior work^21^.

Social distancing measures, such as maintaining a two-meter distance, have been widely recommended to reduce the risk of airborne transmission. However, Issakhov et al.^20^ used CFD simulations to demonstrate that particles expelled during coughing or sneezing can travel up to five meters, suggesting that the WHO’s recommended two-meter distance may be insufficient in certain environments. Their findings emphasize the need for combining social distancing with optimized ventilation strategies to effectively mitigate transmission risks.

The integration of ventilation strategies with social distancing measures has been shown to significantly reduce the risk of airborne transmission. Nie et al.^22^ used transient CFD modeling to evaluate the effectiveness of upward ventilation and face masks in reducing indoor infectious concentrations, finding that combined measures reduced concentrations by 99.95% compared to scenarios without precautions. Their study emphasized the importance of achieving an Air Changes per Hour (ACH) value of 10 to effectively reduce droplet concentrations.

### Droplet evaporation and air distribution design

The evaporation of respiratory droplets is another critical factor influencing airborne transmission. Dao and Kim^23^ studied the behavior of cough droplets in hospital isolation rooms, finding that low relative humidity (RH) accelerates droplet evaporation, leading to the formation of smaller aerosols that can remain airborne for extended periods. Their research highlighted the importance of exhaust vent placement in air conditioning design, with optimal placement achieving 99% droplet removal efficiency within 90 s.

Similarly, Yan et al.^24^ investigated the transmission of COVID-19 in an aircraft cabin, demonstrating that cough-induced particles from window-seat passengers posed the highest exposure risk due to their extensive dispersion within the cabin. Their findings underscore the importance of designs in reducing the spread of airborne pathogens in confined spaces.

While CFD modeling is recognized for its utility in comprehending aerosol dispersion and refining air distribution designs, there is still more to learn about airborne transmission. This study will contribute to that understanding by examining the influence of airflow modulation and specific strategies on respiratory droplet dispersion within office settings.

Using CFD simulations, the paper explores how different configurations, including the placement of supply and exhaust diffusers, influence the spread of aerosols and the effectiveness of social distancing measures. The findings from this research provide quantitative insights into optimizing indoor air management and reducing the risk of airborne transmission, with implications for public health.

Unlike previous research that primarily focuses on airflow patterns or general ventilation efficiency, this study examines how airflow modulation directly influences airborne particle behavior, emphasizing the role of supply and exhaust diffuser placement in controlling aerosol dispersion. Additionally, this study builds upon prior research conducted by the authors, which focused on airflow patterns in the same office environment, previously published by authors previously^9^. This research bridges the gap between ventilation engineering and infection control strategies by expanding its scope to include particle transport analysis. Consequently, its findings are applicable to HVAC system optimization, indoor air quality management, and public health interventions.

This study presents a multidisciplinary CFD-based framework for mitigating airborne transmission. Utilizing an Eulerian–Lagrangian approach integrated with the Discrete Phase Model (DPM), the research extends beyond conventional airflow analysis by incorporating detailed aerosol dynamics. These include droplet transport, evaporation, and dispersion, evaluated under varying ventilation configurations to assess their impact on exposure risk. This research aims to evaluate airborne transmission mitigation strategies by:

- Simulating airflow modulation techniques using CFD with an Eulerian–Lagrangian framework to assess their impact on aerosol dispersion and removal efficiency.
- Applying the Discrete Phase Model (DPM) to track droplet transport, evaporation, and transformation into airborne nuclei, ensuring an accurate representation of aerosolized pathogen behavior.
- Examining different ventilation configurations, particularly the placement of supply and exhaust diffusers, to determine their role in controlling particle dispersion and residence time in indoor office environments.
- Providing a detailed analysis of airflow interactions with expelled particles, demonstrating how ventilation strategies influence aerosol transport and accumulation in enclosed spaces.
- Contributing to HVAC system optimization by offering quantitative insights for designing more effective air distribution systems to minimize airborne contamination risks.

By achieving these aims, this study intends to establish a scientifically grounded understanding of how airflow modulation impacts respiratory particle behavior, with applications in workplace air quality, HVAC system efficiency, and infection control strategies for enclosed environments.

## Materials and methods

This section outlines the comprehensive methodology employed to simulate aerosol dispersion and evaluate ventilation strategies in an indoor office environment. The study begins with a description of the simulation geometry and domain setup, including diffuser configurations and occupant modeling. It then details the numerical methods and simulation parameters used to solve airflow and particle transport using an Eulerian–Lagrangian approach. Subsequent subsections present the mesh generation and refinement strategy, grid independence analysis using Richardson Extrapolation and GCI, and the governing equations of the continuous phase (air) and discrete phase (droplets). The particle injection modeling, including droplet sizing, thermal properties, evaporation dynamics, and turbulence dispersion, is discussed in detail. The section concludes with validation procedures comparing simulation outputs to established literature and experimental benchmarks. Together, these elements ensure the robustness, accuracy, and physical fidelity of the simulation framework.

### Simulation geometry and setup

**Fig. 1:**
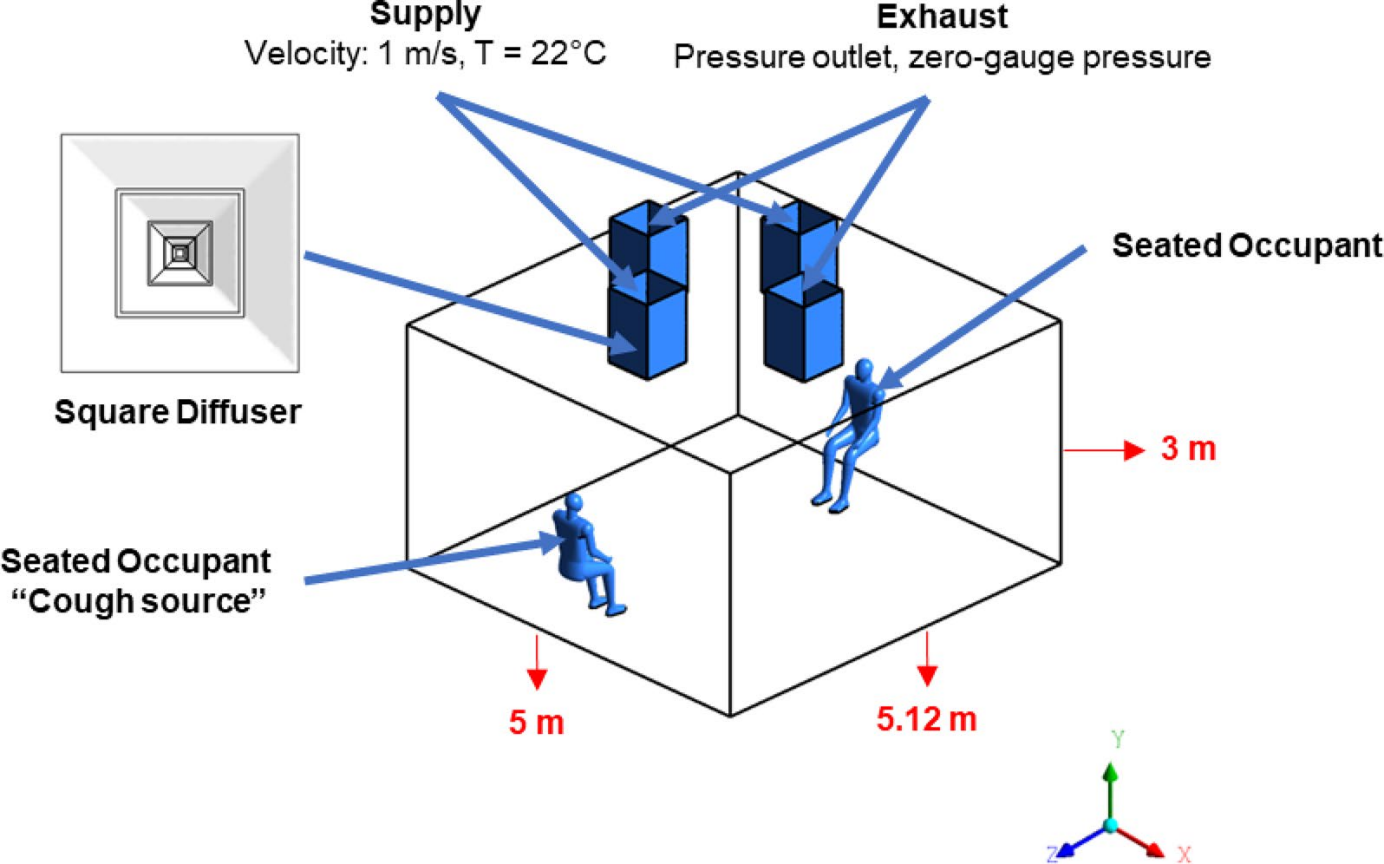
Three-dimensional model of the office environment with seated human mannequins. Room dimensions: 5.12 m (length) × 5.00 m (width) × 3.00 m (height). Supply diffusers (velocity inlet: 1 m/s at 22 °C) and exhaust diffusers (pressure outlet) are shown for both simulated cases. The occupant marked as “cough source” represents the aerosol emission location.

**Fig. 2:**
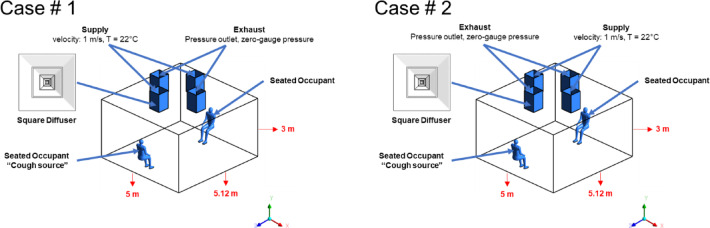
Ventilation layouts for Case #1 (central exhaust, perimeter supply) and Case #2 (exhaust above cough source, central supply), used to assess the effect of diffuser positioning on aerosol dispersion.

The simulation will take place in a workplace located in Alexandria, Egypt with an already tested and published research for airflow in various air ceiling diffusers^9^. The physical model used in this study represents office space with specific dimensions. The office has a height of 3 m (y), a width of 5 m (x), and a length of 5.12 m (z) (Fig. 1) provides a three-dimensional visual representation of the room being analyzed, illustrating the positions of employee mannequins within the space. The human sitting mannequin is 1.5 m sitting height with a 320 mm^2^ mouth opening, the room setting as illustrated in showing two sitting human bodies. The coughing personnel will be sitting in the room marked as the cough source (Fig. 1). In this model, the square air diffuser was used in simulation (Fig. 1). The model development and simulation were carried out using ANSYS Fluent (Version 19.1), a widely validated computational tool capable of performing both qualitative and quantitative analyses of airflow and heat transfer in enclosed environments. The existing HVAC system^9^ served as the foundation for this model. The three-dimensional (3D) geometry of the model was created using ANSYS-Design Modeler, providing precise control over the placement of diffusers. The numerical setup in this study is adapted for two different air conditioning configurations, aiming to analyze how airflow patterns influence the transport and dispersion of cough-generated droplets within an office environment, and their potential role in increasing infection risk. To examine so, the Exhaust diffusers will be replaced by supply diffusers and vice versa as shown in Fig. 2, contrasting the effect of the parallel exhaust diffusers in the center of the workplace (Case # 1) to the location of the exhaust diffuser above the staff sitting in the office (Case # 2).

### Numerical setup and simulation parameters

To increase the study’s comprehensiveness, researchers assess exposure dangers using aerosol concentration as a parameter. Indoor airflow and droplet transmission are simulated using an Euler-Lagrangian model. While the droplets that people exhale are seen to be separate phases, the air is thought to be a continuous phase. The simulation technique may be loosely separated into two primary parts. The Lagrangian model is used to predict the trajectory of cough droplets after the time-dependent Euler model has solved interior flow fields. Moreover, this simulation anticipates droplet evaporation by considering mass and heat transport between droplets and the surrounding air^25,26^.

**Fig. 3:**
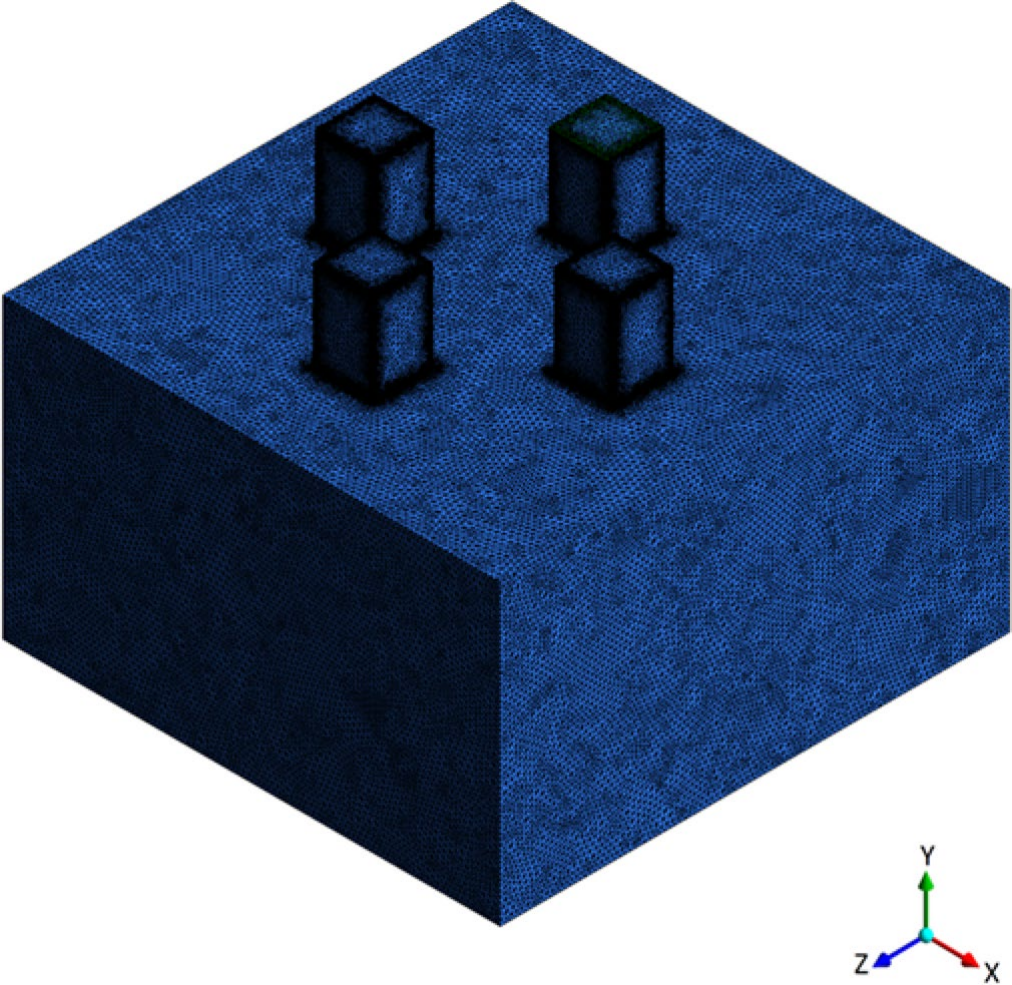
Computational domain with hexahedral mesh. Room dimensions are 5.12 m × 5.00 m × 3.00 m.

ANSYS Meshing was employed to generate the computational grid and proved to be a reliable tool for achieving high-quality mesh control. The mesh (Fig. 3) was constructed based on the actual geometric proportions of the office layout, as depicted in Fig. 1 ensuring geometric fidelity between the simulation domain and the real-world environment.

**Fig. 4:**
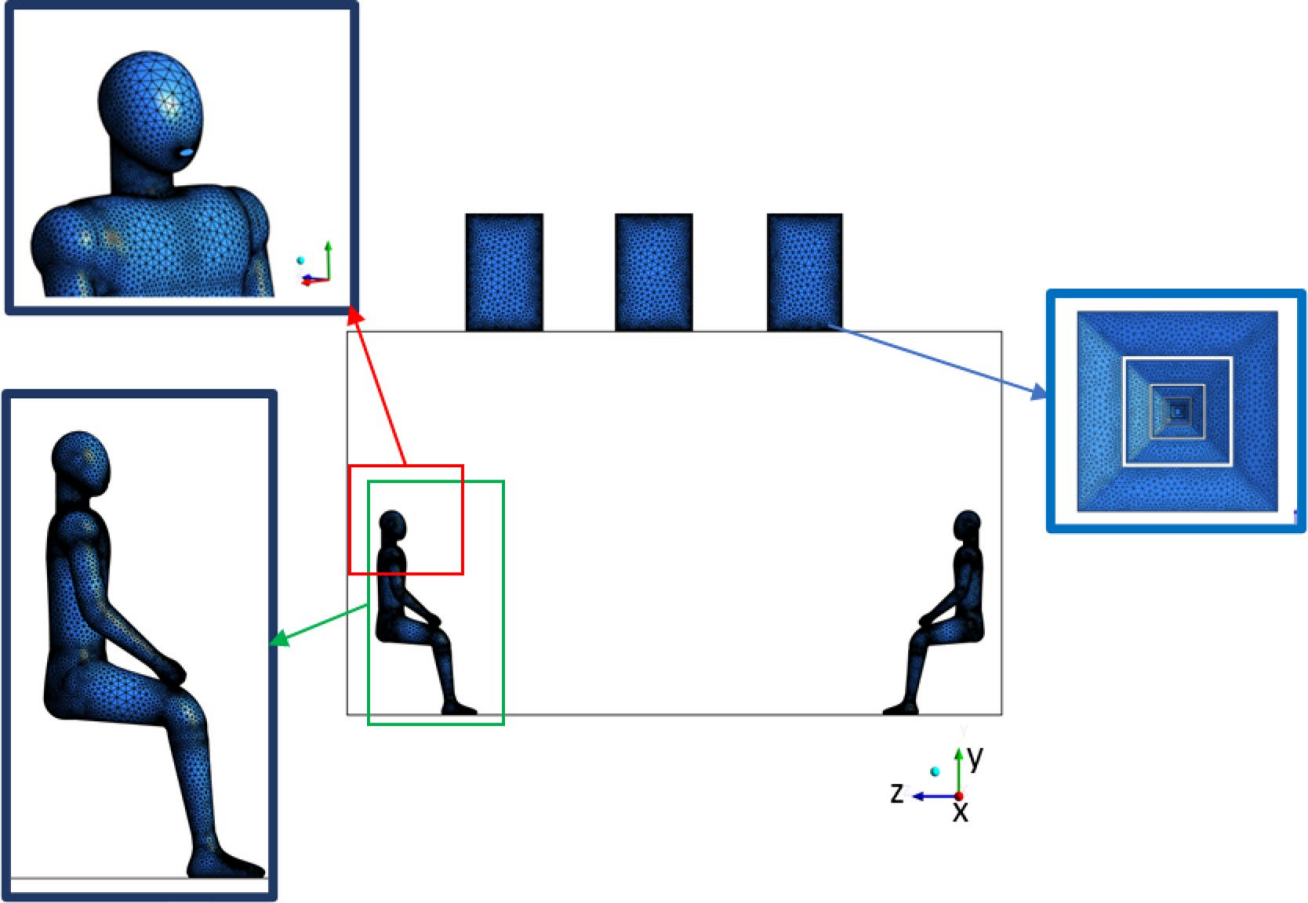
Local mesh refinement zones. Insets show fine mesh at the occupant’s face, body, and ceiling diffuser.

To improve local accuracy and better resolve flow features, targeted mesh refinement was applied in critical regions, as shown in Fig. 4, including:

- The mouth opening of the coughing mannequin, where sharp velocity gradients and droplet injection occur.
- The vicinity of supply and exhaust diffusers, where directional airflow and turbulence are most prominent.
- The surface of the human body, where recirculation zones and droplet deposition are likely.

These refinements allowed for accurate resolution of airflow and droplet dynamics in key areas of interest, thereby enhancing the physical realism and reliability of the simulation results.

ANSYS Fluent was utilized to perform the simulations based on the Eulerian–Lagrangian framework, due to its comprehensive suite of boundary condition options and advanced physics models capable of accurately representing heat transfer and fluid flow. The simulation setup included the specification of velocity inlets, pressure outlets, and wall boundaries, along with the definition of fluid properties such as viscosity, density, and thermal conductivity. All enclosing surfaces, walls, ceiling, and floor were modeled as adiabatic boundaries, implying that no heat flux was exchanged through these surfaces. This assumption allowed the simulation to maintain thermally isolated conditions, facilitating a more controlled assessment of airflow behavior and droplet dispersion within the indoor environment.

Boundary conditions were retrieved from the previously published work by authors^9^ to set up simulation in the ANSYS-FLUENT application, the conditioned fresh air was introduced to the supply grilles as 1 m/s inlet velocity and 22 °C to as a low-speed setting for the air conditioning unit. The exhaust had an outflow zone set. The human body’s surface temperature, measured at 32 °C^27^. At the supply diffusers (velocity inlets), turbulence intensity and hydraulic diameter were specified as 5% and 0.5 m, respectively. Exhaust diffusers (Pressure outlets) were treated with zero-gauge backpressure and backflow turbulence intensity of 5%. All solid surfaces were treated as no-slip, adiabatic walls with enhanced near-wall treatment applied to resolve boundary layer effects.

To ensure accurate near-wall treatment, the mesh was refined such that the dimensionless wall distance (y⁺) values remained predominantly in the range of 1 < y⁺ < 5, which is within the recommended bounds for the use of enhanced wall treatment^28^ in combination with the standard k-ε turbulence model. This resolution allows the solver to adequately resolve the viscous sublayer and ensures fidelity in capturing near-wall flow phenomena. The second-order upwind scheme is employed to discretize the equations related to momentum, energy, k, and $\varepsilon$. To accurately capture the pressure gradient at the boundary, the "PRESTO!" scheme is used to discretize the pressure term. The algebraic equations are solved using a coupled algorithm.

The transient simulation was conducted over a duration of 10 s, which was selected to capture the initial dispersion phase of respiratory droplets following a cough event, an interval known to encompass the highest exposure risk due to concentrated particle release and dominant airflow effects. This approach is consistent with similar transient CFD studies in the literature^29,30^, which analyzed airborne droplet dynamics within comparable timeframes. While longer simulations may provide insights into long-term mixing or viral decay, the present study focuses on early-stage aerosol transport and ventilation influence, which are critical for evaluating exposure risk and mitigation strategies.

A time step of 0.01 s was selected to adequately resolve the highly transient dynamics of the cough jet and droplet injection, which exhibit rapid acceleration and turbulent mixing within the first second of release. This time resolution ensures accurate capture of flow development and particle trajectories without compromising numerical stability. The selected time step is consistent with prior studies^29,31^ on respiratory events, which employed similar temporal resolutions for modeling cough-induced airflow and aerosol dispersion. A time step sensitivity check was conducted by comparing simulations with values of 0.005 s, 0.01 s, and 0.02 s. The results showed less than 2% variation in key parameters such as peak cough jet velocity and particle travel distance, confirming that the selected time step of 0.01 s offers an appropriate balance between temporal accuracy and computational efficiency.

The convergence criterion for the simulation is defined as the energy relative residual reaching 10^–7^ and the relative residuals of other variables reaching 10^–4^. Convergence is typically achieved with 20 iterations for each timestep.

### Mesh sensitivity and grid independence analysis

To evaluate numerical accuracy and eliminate discretization-related uncertainties, a mesh sensitivity analysis was performed using three systematically refined grids comprising approximately 0.3 million, 1.2 million, and 4.8 million hexahedral cells. These grids were generated with consistent refinement ratios to maintain geometric and topological integrity across simulations. The analysis was centered on a representative monitored quantity specifically, the peak velocity magnitude in the vicinity of the cough jet source chosen for its sensitivity to mesh resolution. The mesh sensitivity of the numerical solution was assessed using the Richardson Extrapolation method and the Grid Convergence Index (GCI), following the guidelines proposed by Roache (1999)^32^, to ensure the reliability and mesh-independence of the simulation results.

The peak velocity results obtained were 16.3 m/s, 16.0 m/s, and 15.9 m/s for the coarse, medium, and fine meshes, respectively. The apparent order of convergence ($p$) was calculated as 0.79, using Richardson Extrapolation:

1 $$p = \frac{{\ln \left( {\frac{{\phi_{3} - \phi_{2} }}{{\phi_{2} - \phi_{1} }}} \right)}}{\ln \left( r \right)}$$

where ${\phi }_{1}$, ${\phi }_{2}$, and ${\phi }_{3}$ represent the monitored quantity (peak velocity) on the fine, medium, and coarse grids, respectively, and $r$ is the uniform grid refinement ratio. Once the order of convergence was obtained, the Richardson Extrapolated Value ${\phi }_{ext}$ was computed as:

2 $$\phi_{ext} = \phi_{1} + \frac{{\phi_{1} - \phi_{2} }}{{r^{p} - 1}}$$

This provided an estimate of the solution on an infinitely fine grid, yielding ${\phi }_{ext}$ = 15.85 m/s. Furthermore, the Grid Convergence Index (GCI) was calculated for the finest mesh as:

3 $$GCI_{fine} = \frac{{1.25\left| {\phi_{1} - \phi_{2} } \right|}}{{\left| {\phi_{1} } \right|(r^{p} - 1)}} \times 100\%$$

The resulting GCI value of 0.39% confirms that the numerical solution is mesh-independent and within an acceptable margin of discretization error for engineering accuracy.

**Fig. 5:**
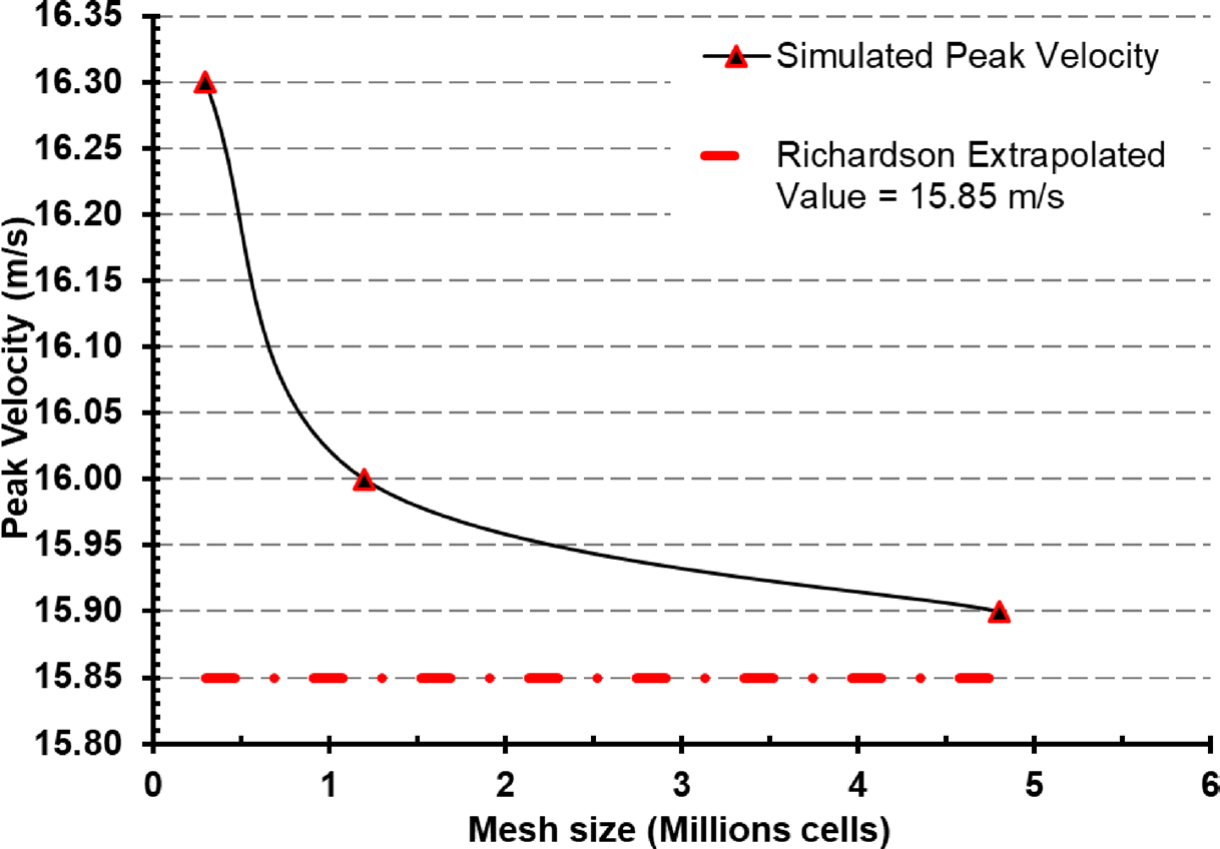
Mesh sensitivity and grid independence analysis with mesh size vs peak velocity.

Based on this evaluation, the 4.8 million-cell mesh was selected for all subsequent simulations, as it offered a balance between computational cost and solution fidelity. Figure 5 presents the convergence behavior of peak velocity as a function of mesh resolution, along with the extrapolated reference value.

### Euler model (air model)

As mentioned earlier, the simulation process is divided into two phases. The first phase is the flow of air model. In this study, the standard k-ε turbulence model was utilized to simulate the airflow field within the computational domain along with the enhanced wall treatment^33^. The Reynolds-Averaged Navier–Stokes (RANS) equations were employed to model the mean flow behavior, while turbulence effects were captured using the two-equation k-ε model^34^, which has been extensively validated for applications in indoor airflows and ventilation studies. The governing equations for incompressible turbulent flow are given as follows:

- The continuity equation:
  4 $$\frac{\partial \rho }{{\partial t}} + \nabla \cdot \left( {\rho {\text{u}}} \right) = 0$$
- The momentum equation:
  5 $$\frac{{\partial \left( {\rho {\text{u}}} \right)}}{\partial t} + \nabla \cdot \left( {\rho {\text{uu}}} \right) = - \nabla P + \nabla \cdot \left( {\mu_{{{\text{eff}}}} \nabla {\text{u}}} \right) + \rho {\text{g}}$$

where $\rho$ is air density, $\text{u}$ is velocity, $P$ is pressure, and ${\mu }_{\text{eff}}$​ is the effective viscosity.

The standard k-ε model introduces two additional transport Eqs. ^34^ to model turbulence:

- The turbulent kinetic energy (k) equation:
  6 $$\frac{\partial }{{\partial {\varvec{t}}}}\left( {\rho \kappa } \right) + \frac{\partial }{{\partial {\varvec{x}}_{i} }}\left( {\rho \kappa {\varvec{u}}_{i} } \right) = \frac{\partial }{{\partial {\varvec{x}}_{j} }}\left( {{\Gamma }_{k} \frac{\partial \kappa }{{\partial x_{j} }}} \right) + {\varvec{G}}_{{\varvec{k}}} + {\varvec{G}}_{b} - \rho \varepsilon - {\varvec{Y}}_{{\varvec{M}}} + {\varvec{S}}_{{\varvec{k}}}$$
- The turbulent dissipation rate (ε) equation:
  7 $$\frac{\partial }{{\partial {\varvec{t}}}}\left( {\rho \kappa } \right) + \frac{\partial }{{\partial {\varvec{x}}_{i} }}\left( {\rho \kappa {\varvec{u}}_{i} } \right) = \frac{\partial }{{\partial {\varvec{x}}_{j} }}\left( {{\Gamma }_{k} \frac{\partial \kappa }{{\partial {\varvec{x}}_{j} }}} \right) + {\varvec{G}}_{{\user2{I\varepsilon }}} \frac{{\varvec{\varepsilon}}}{{\varvec{\kappa}}}\left( {{\varvec{G}}_{{\varvec{k}}} + {\varvec{G}}_{3\varepsilon } {\varvec{G}}_{b} } \right) + {\varvec{G}}_{2\varepsilon } \rho \frac{{{\varvec{\varepsilon}}^{2} }}{{\varvec{\kappa}}} + {\varvec{S}}_{\varepsilon }$$

where *G*_*k*_ and *G*_*b*_ are the kinetic energy of turbulence produced by the mean velocity gradients and buoyancy and *Y*_*M*_ The variable dilatation of compressible turbulence contributes to the total dissipation rate. As for *G*_*1ε*_,* G*_*2ε*_ and *G*_*3ε*_ considered as constants.

The standard k-ε turbulence model was selected in this study due to its well-documented applicability in indoor airflow modeling and its proven performance in mechanically ventilated environments. This model offers a computationally efficient means of resolving large-scale turbulent structures, making it particularly suitable for room-scale simulations of airflow and aerosol dispersion. Given the study’s focus on evaluating ventilation configurations and the transport of airborne particles in a high-flow indoor setting, the k-ε model provides a practical balance between numerical accuracy and computational cost. Previous studies^35–38^ have shown that the k-ε model performs adequately in predicting airflow distribution and contaminant transport under similar conditions. Moreover, the validation of airflow fields and droplet dispersion patterns in “HVAC and cough particle validation” section confirms that the simulated results align well with experimental observations, supporting the reliability of the turbulence modeling approach used.

Nevertheless, we acknowledge that alternative Reynolds-Averaged Navier–Stokes (RANS) models may offer enhanced accuracy, particularly for capturing flow separation, near-wall effects, and anisotropic turbulence. Models such as the realizable k-ε, RNG k-ε, and especially the SST k-ω have been shown to outperform the standard k-ε in complex geometries or scenarios involving particle deposition. For instance, Gao and Niu^39^ demonstrated that SST k-ω yields more accurate predictions of particle deposition in turbulent channel flows. Similarly, Zabihi et al.^40^ successfully employed Euler–Lagrange URANS simulations to capture the transient dispersion of aerosols indoors, highlighting the benefit of refined turbulence closure models. Ai and Melikov^41^ further advocate for advanced turbulence modeling when studying exposure in proximity to contaminant sources. While the standard k-ε model remains appropriate for the current room-scale evaluation in a mechanically mixed environment, future work may incorporate these advanced models to better resolve localized airflow phenomena, near-wall dynamics, and model sensitivity in more complex or stratified indoor conditions.

### Particle transportation and sizing (cough model)

As for the lagrangian model (cough model), the focus is on examining the droplets released during coughing. Data related to cough measurements were adapted from Experimental benchmark by Duguid^42^, that conducted measurements of the size distribution of cough droplets using a microscope. It was reported that the velocity of the expelled airflow during coughing is approximated by combining multiple sine functions, based on experimental data obtained from a spirometer that utilized a Fleish type pneumotachograph. The maximum velocity of the airflow is recorded as 15.9 m/s.

**Fig. 6:**
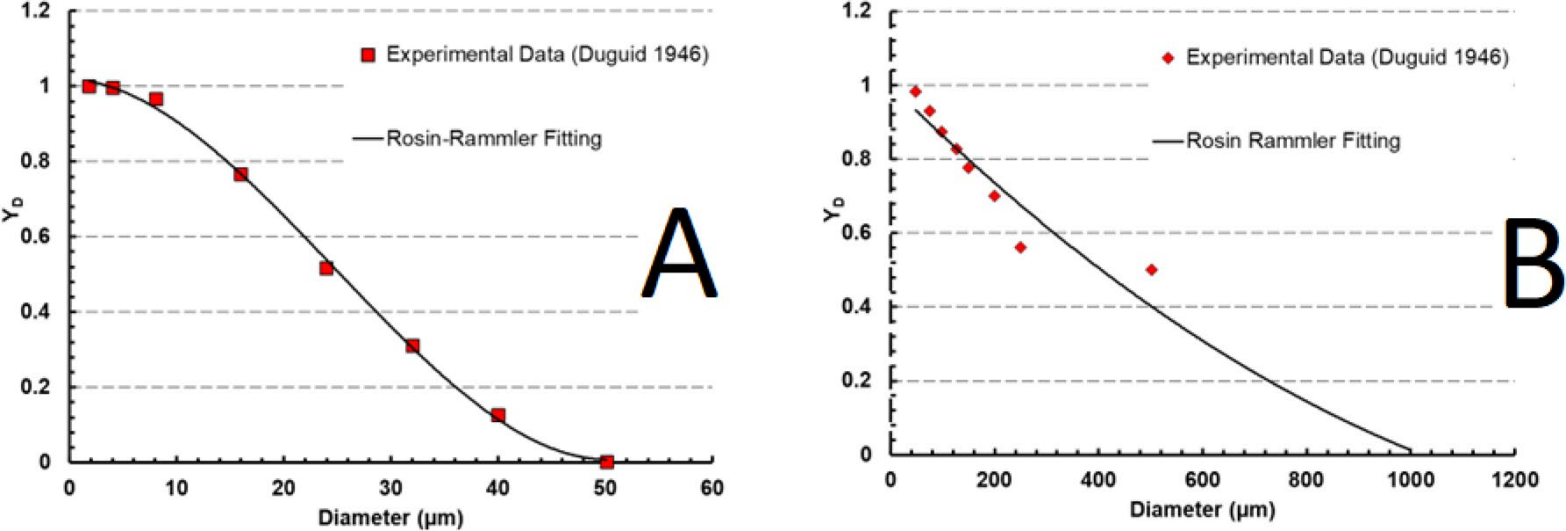
Rosin–Rammler fitting for cough particles diameters varying into two set of sizes. (**A**) 1–50 μm. (**B**) 50–1000 μm.

Droplets released by coughing range in size from 1 to 1000 μm. We make use of two Rosin–Rammler distributions to fit the droplet diameter distributions of 1 to 50 μm and 50 to 1000 μm (Fig. 6), respectively, due to the variation of cough droplets diameter. ${Y}_{d}$ is labelled in the Rosin–Rammler distribution function as the mass fraction of droplets having a diameter larger than $d$. Furthermore, according to Rosin and Rammler^43^, there is an exponential connection between ${Y}_{d}$ and $d$. Va

8 $$Y_{d} = e^{{ - \left( {\frac{d}{{\overline{d}}}} \right)^{n} }}$$

where $\overline{d }$ represents the mean droplet diameter, and $n$ denotes the spread parameter, assigned values are represented in Table 1. Solid cone injection method was utilized in front of the mouth weighing 7.7 mg^44,45^. Injection duration was 0.5 s with a total of 35,000 parcels. According to the overall mass and Rosin–Rammler distribution, each parcel reflects a certain number of droplets.

**Table 1:** Rosin–Rammler parameters (n and $\overline{\text{d} }$) for Fig. 6A and B.

Figure	Mean diameter ($\overline{d })$	Spread parameter (n)
Figure 6A	20 μm	2.9
Figure 6B	15 μm	3.1

The angle between the cough airflow and the horizontal is 27.5°, and the droplet parcel injection velocity is 6.6 m/s^29,46^. The path that cough droplets take is examined using the discrete phase model (DPM). The non-steady motion of individual droplet packets is seen by the application of Newton’s second law and the consideration of the effects of drag force, gravity, and buoyancy^47,48^. According to Nicas et al.^49^, the concentration of non-volatile components in cough droplets is 1.8%, meaning that the equilibrium diameter of the droplet nucleus is approximately 26% of its initial size. The particles were not handled in bulk; instead, each droplet parcel represented a group of droplets, ensuring computational efficiency while maintaining accurate dispersion dynamics. The injected droplets were assigned an initial temperature of 310.15 K (37 °C), representing the average temperature of exhaled respiratory fluid.

To accurately model the behavior of airborne droplets emitted from coughing, a Eulerian–Lagrangian approach was employed, where the airflow field was simulated using the Reynolds-Averaged Navier–Stokes (RANS) equations with a k-ε turbulence model^28^, and individual droplet trajectories were tracked using the Discrete Phase Model (DPM) in ANSYS Fluent. The path of cough droplets was investigated using the discrete phase model (DPM). Using Newton’s second law and accounting for the effects of drag force, gravity, and buoyancy, each droplet parcel is observed for unstable motion^50^

9 $$\frac{{d\overrightarrow {{u_{p} }} }}{dt} = \frac{{\vec{u} - \overrightarrow {{u_{p} }} }}{{\tau_{r} }} + \frac{{\vec{g}\left( {\rho_{p} - \rho } \right)}}{{\rho_{p} }}$$

where $\overrightarrow{{u}_{p}}$ is the droplet velocity, ${\tau }_{r}$ is the droplet relaxation time, which depends on the diameter and density of the droplet, $\overrightarrow{g}$ is the gravity acceleration, and $\rho$ is the droplet density.^48^.

The simulation accounted for multiple physical mechanisms influencing airborne particle motion, ensuring an accurate representation of aerosol transport. Gravity and buoyancy effects were incorporated, where larger droplets experienced rapid gravitational settling, while smaller aerosols remained suspended due to their interaction with turbulent airflow patterns. Turbulent dispersion was modeled using the Random Walk Model (Stochastic Tracking Approach), allowing particle trajectories to fluctuate in response to local airflow variations^51^.

10 $$u_{i} = \overline{{u_{i} }} + \zeta \sqrt{\frac{2k}{3}}$$

where $\zeta$ is a normally distributed random number, and $k$ is the turbulent kinetic energy.

The eddy interaction time^51^ used in the turbulence dispersion model was defined as

11 $$\tau _{{eddy}} = \frac{{0.15 \cdot k}}{ \in }$$

where $k$ is the local turbulent kinetic energy, and $\epsilon$ is the local rate of dissipation of turbulence.

This formula ensures that particle dispersion responds dynamically to local turbulence levels, with higher turbulence resulting in shorter-lived eddies and greater particle fluctuation. This interaction time governs the lifetime of an eddy before a droplet encounters a new eddy, simulating the effect of turbulent diffusion.

A two-way coupling strategy was employed to allow momentum and energy exchange between the droplet and airflow phases. This approach is particularly suitable for transient events with high local particle concentrations, such as coughing, and has been adopted in similar respiratory droplet studies^52^. The Taylor Analogy Breakup (TAB) model was activated in the DPM setup to account for secondary droplet breakup caused by aerodynamic deformation during the cough event, enhancing the accuracy of droplet size evolution in turbulent airflow.

#### Evaporation handling and phase change

To accurately simulate droplet evaporation, the DPM evaporation model^28^ was employed, where mass transfer was calculated based on latent heat exchange, surrounding air temperature, and relative humidity levels. The mass transfer of water from droplets to the surrounding air was governed by the following equation:

12 $$\dot{m} = - k_{m} A\left( {C_{s} - C_{\infty } } \right)$$

where $m\dot{}$ is the mass evaporation rate (kg/s), ${k}_{m}$ is the mass transfer coefficient (m/s), $A$ is the droplet surface area (m^2^), ${C}_{s}$ is saturation vapor concentration at the droplet surface (kg/m^3^) and ${C}_{\infty }$ is the ambient vapor concentration (kg/m^3^).

The mass transfer coefficient (${k}_{m}$) was determined using the Sherwood number correlation, given by:

13 $$k_{m} = \frac{Sh \cdot D}{d}$$

where $Sh$ is Sherwood number determined by Ranz-marshall correlation for droplet evaporation, $D$ is the binary diffusion coefficient of water vapor in air (m^2^/s) and $d$ is the droplet diameter.

The heat exchange between the droplet and the surrounding air was modeled using the heat balance equation:

14 $$\dot{m}h_{v} = hA\left( {T_{\infty } - T_{d} } \right)$$

where ${h}_{v}$​ is the latent heat of vaporization (J/kg), $h$ is the convective heat transfer coefficient (W/m^2^K), ${T}_{\infty }$ is the ambient air temperature (K) and ${T}_{d}$ is the droplet temperature (K).

This model ensures that droplet size continuously decreases due to evaporation, leading to the formation of residual airborne droplet nuclei that can remain suspended for extended periods. The evaporation rate was influenced by temperature, humidity, and airflow conditions, accurately replicating real-world aerosol transformation dynamics. The numerical implementation of this model allowed for precise tracking of droplet lifetime, helping to assess the risk of airborne pathogen transmission in indoor office environments. Surface interaction dynamics were also considered, with walls, floors, ceilings, and human surfaces modeled using a trap boundary condition, meaning deposited droplets adhered upon contact. On the other hand, supply and exhaust vents were assigned escape boundary conditions, allowing airborne particles to exit the computational domain when entrained within the airflow pathways. By integrating these transport and deposition mechanisms, the model effectively captured the complex behavior of respiratory droplets, including their suspension, evaporation, and surface interactions, ensuring a realistic assessment of airborne pathogen transmission in office environments.

#### Influence of thermal plumes and respiratory cycles on aerosol dispersion

In indoor environments, aerosol dispersion is governed by a combination of factors, including mechanically induced airflow, body-generated thermal plumes, and respiratory flow cycles. While the present study focuses on aerosol dynamics driven by mechanical ventilation, it is important to contextualize the role of thermal plumes and inhalation–exhalation cycles based on current scientific understanding. Thermal plumes arise from convective heat transfer between the human body and the surrounding air, generating upward buoyant flows that may affect the transport of airborne particles in the immediate vicinity of an occupant. However, the influence of these plumes is strongly dependent on the ambient airflow conditions. Several studies have demonstrated that under low-velocity ventilation regimes such as displacement or natural ventilation thermal plumes can significantly impact the direction and concentration of aerosol transport near the breathing zone. For instance, Zong et al.^53^ highlighted that thermal plumes increase exposure risk by drawing particles from lower regions into the breathing zone when background airflow velocities are below approximately 0.25 m/s.

Conversely, in mechanically ventilated environments with higher supply airflow velocities, the effect of thermal plumes is substantially diminished. Yang et al.^54^ showed that ventilation velocities at or above 0.25 m/s are sufficient to disrupt thermal plumes and limit their influence on overall airflow patterns. Similarly, Ai and Melikov^41^ concluded that in well-mixed indoor environments driven by mechanical ventilation, forced convection dominates particle transport, thereby minimizing the contribution of buoyancy-driven flows. These findings are further supported by Zabihi et al.^55^, who demonstrated through CFD simulations that while thermal plumes can elevate particles locally, the broader dispersion and removal of aerosols are primarily governed by mechanical ventilation. Given that the airflow velocities modeled in our study exceed these critical thresholds, the exclusion of thermal plumes is justified and consistent with established literature.

The continuous inhalation–exhalation cycles of occupants also play a role in shaping airflow fields and particle transport, particularly in short-range transmission scenarios. However, their influence is largely confined to the near-field region (within approximately 1 m of a source), as shown by Zong et al.^53^ and others. Although prior studies have incorporated respiration dynamics into CFD models, their impact becomes less significant at the room scale under active mechanical ventilation. In this study, a discrete high-momentum cough event is used as the representative emission source to evaluate aerosol transport across space. Given the focus on room-scale ventilation performance rather than close-contact exposure, the exclusion of breathing cycles is considered appropriate for the study objectives.

Although thermal plumes and respiratory cycles may affect localized aerosol dynamics under specific conditions, their impact is considerably reduced in high-flow, mechanically ventilated environments such as the one examined in this work. Given the elevated airflow velocities modeled well above thresholds where buoyancy-driven or respiratory effects become significant, their exclusion is unlikely to alter the overall findings related to ventilation strategies and aerosol dispersion. Nevertheless, their importance in proximity-based transmission and stratified airflow conditions is acknowledged, and future studies may incorporate these factors to expand the applicability of airflow and exposure assessments to a broader range of indoor environments.

### HVAC and cough particle validation

The validation process was conducted in three sequential stages to ensure the accuracy and reliability of the simulation results. First, the HVAC airflow field was validated based on previously published experimental data from past research^9^, where simulated airflow patterns were compared with on-site velocity measurements. Second, the cough jet dynamics were validated by comparing the simulated trajectory and behavior of the cough cloud with experimental data on human expiratory events. Finally, the droplet transport and evaporation behavior were validated using two references: the Wells evaporation–falling curve for assessing vertical displacement trends, and Ugarte-Anero et al. (2022) for time-resolved diameter decay, confirming the evaporation and dispersion accuracy over the 10-s simulation period.

**Fig. 7:**
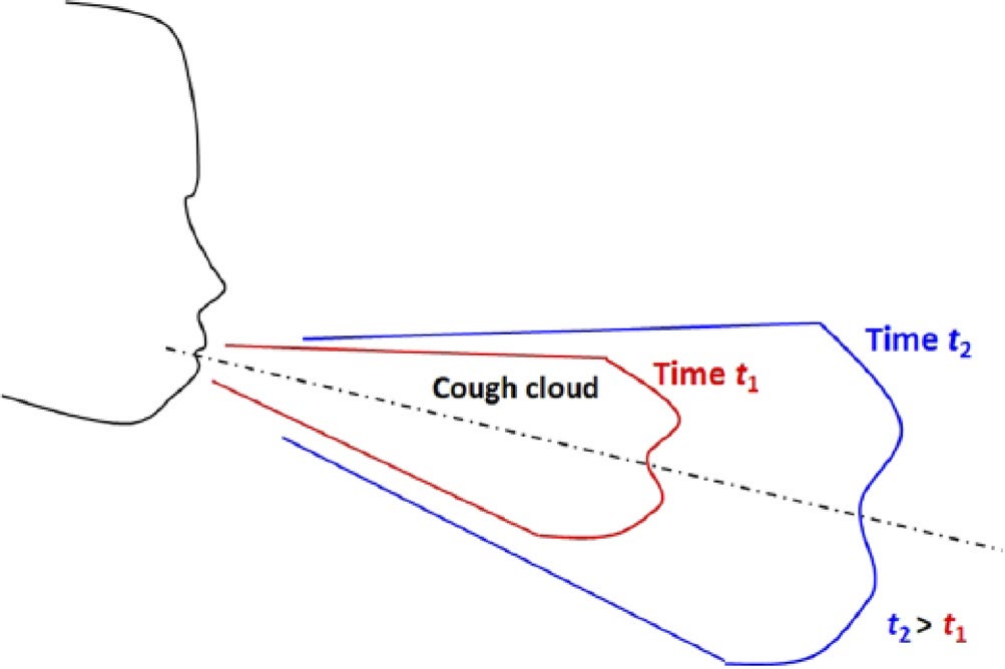
Schematic of the cough cloud generated by a human being^56^.

The DPM model has received extensive validation, performing well in forecasting droplet mobility and deposition. Nonetheless, the velocity of air greatly affects how droplets travel. Prior to examining the droplet lifespan released during a cough, we confirm the cough airflow phase’s validity. Agrawal and Bhardwaj developed a mathematical formulation for predicting the distance traveled by a cough cloud in an enclosed area, building upon experimental findings^56^. The validation process was conducted by tracking the movement distance of the cough cloud front (Fig. 7), utilizing 1 μm diameter particles to estimate the advancement of the cough cloud over time. These particles were expelled from the mouth and closely followed as they interacted with the surrounding airflow, ensuring accurate representation of the cloud’s dispersion dynamics.

**Fig. 8:**
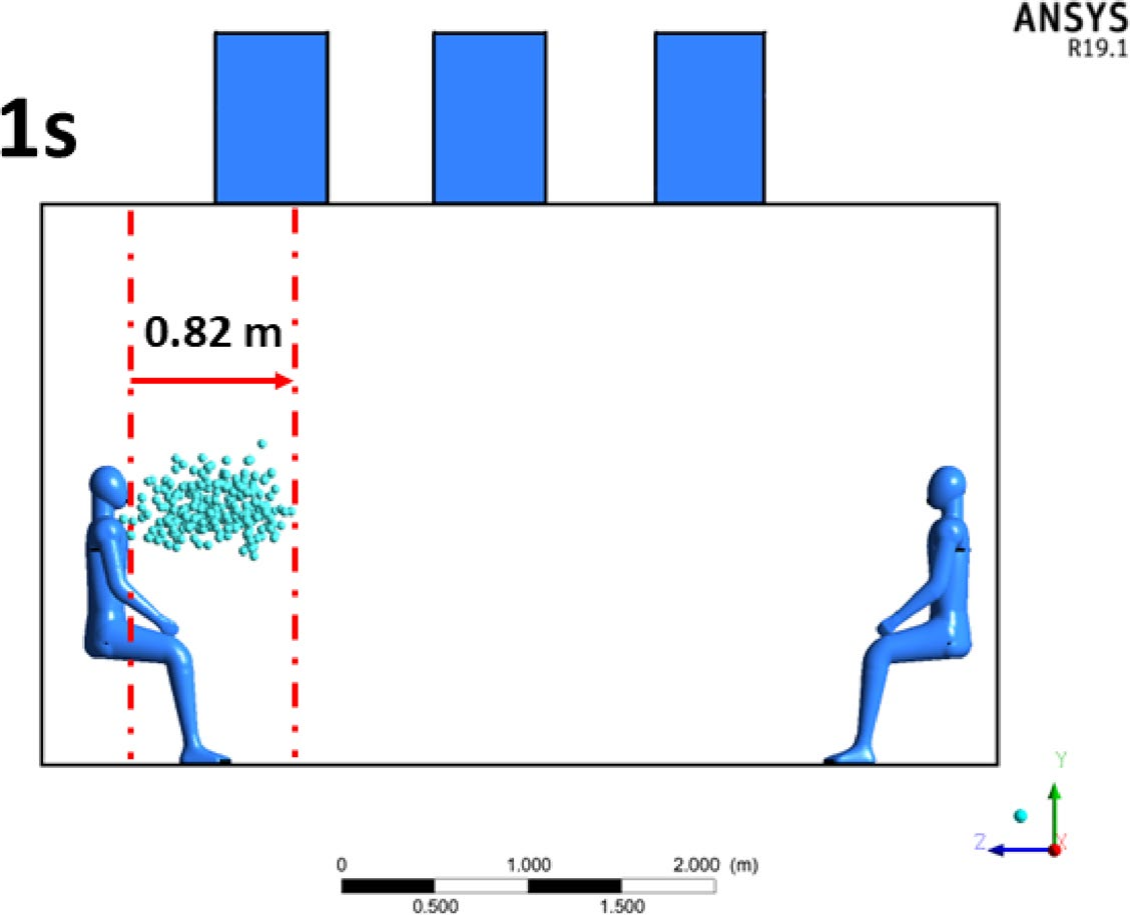
Cough cloud dispersion in 1 s of simulation (current study),created using ANSYS R19.1 (https://www.ansys.com).

Figure 8, a representative snapshot of the simulated cough cloud is presented, illustrating particle dispersion at 1 s and capturing the cloud measurement process over a 10-s timeline for validation purposes. The cloud was measured from the point of expulsion (mouth region) to the farthest detected particle, ensuring a comprehensive evaluation of its propagation.

**Fig. 9:**
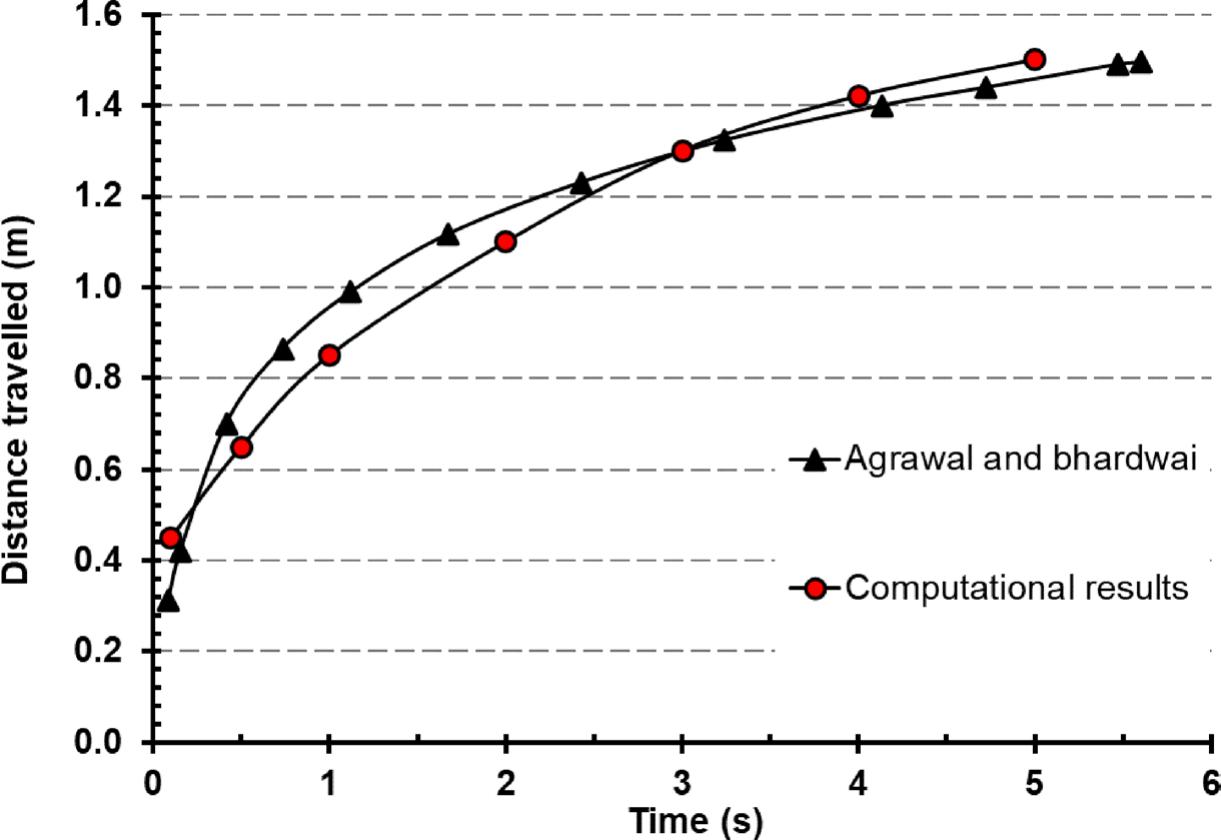
Distance of cough cloud change with time (Agrawal and Bhardwai^57^ vs computational results (current study)).

The validation results demonstrated strong agreement between the computational model and the mathematical reference model (Fig. 9). The maximum observed variation between the simulation and the mathematical model^56^ was 11%, indicating a high level of accuracy in the numerical approach. Furthermore, the discrepancy between the models decreased significantly after 3 s, suggesting that the CFD simulation effectively captures the transient behavior of the cough cloud as it stabilizes over time. These findings reinforce the reliability of the current model in accurately predicting airborne particle transport and dispersion.

**Fig. 10:**
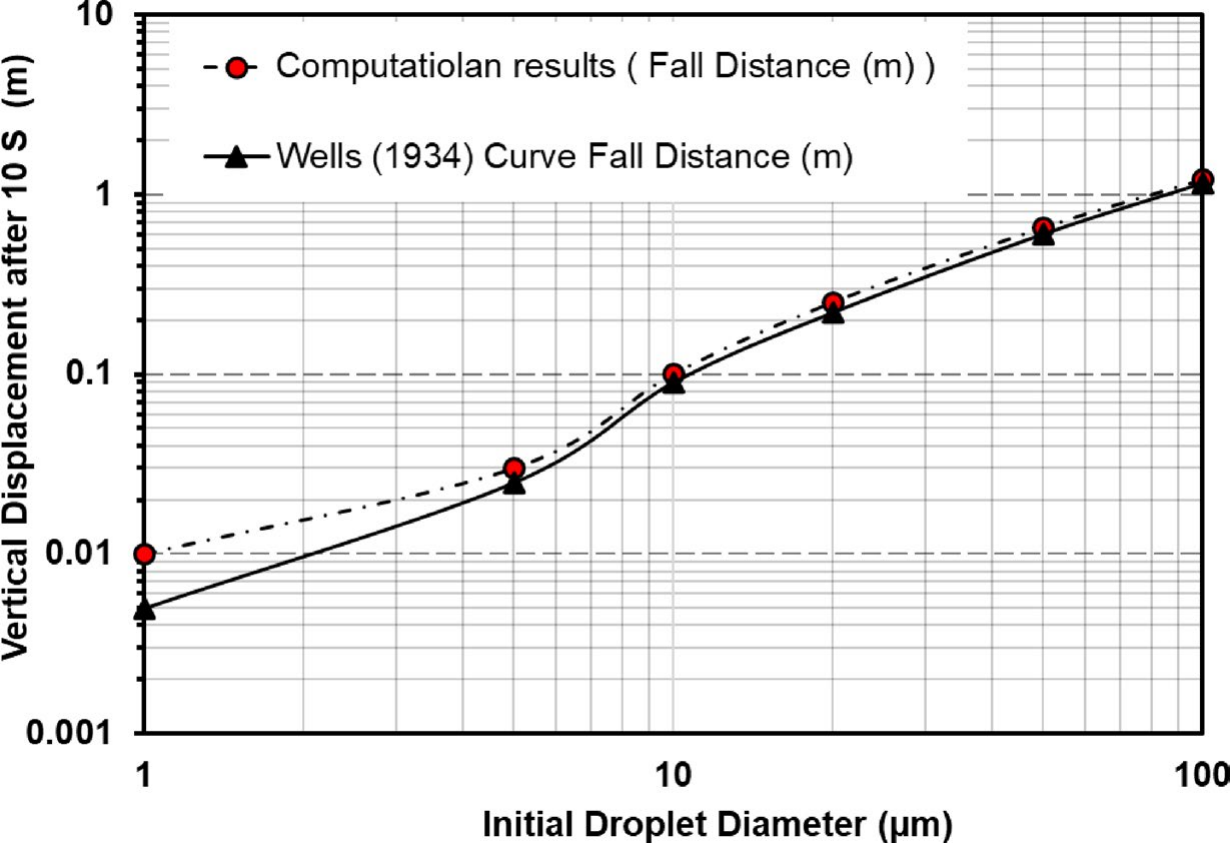
Validation of droplet vertical displacement against the Wells evaporation–falling curve^58^.

To further validate the accuracy of the droplet dynamics modeled in this study, the simulated vertical displacement of droplets over a 10-s period was compared against the classical Wells evaporation–falling curve, originally proposed by Wells (1934)^58^. The Wells curve outlines the relationship between droplet size and settling behavior in indoor environments, highlighting that smaller droplets (< 10 μm) remain suspended due to rapid evaporation and air resistance, while larger droplets (> 50 μm) tend to settle quickly under gravity. In our simulation, representative droplets with initial diameters of 1, 5, 10, 20, 50, and 100 μm were tracked using the Discrete Phase Model (DPM). Their vertical displacement was recorded after 10 s, and the results were compared with scaled reference values derived from the Wells curve. As shown in Fig. 10, the simulation results exhibited good agreement with the theoretical trend: larger droplets displayed significant downward motion, while smaller droplets showed minimal displacement.

**Fig. 11:**
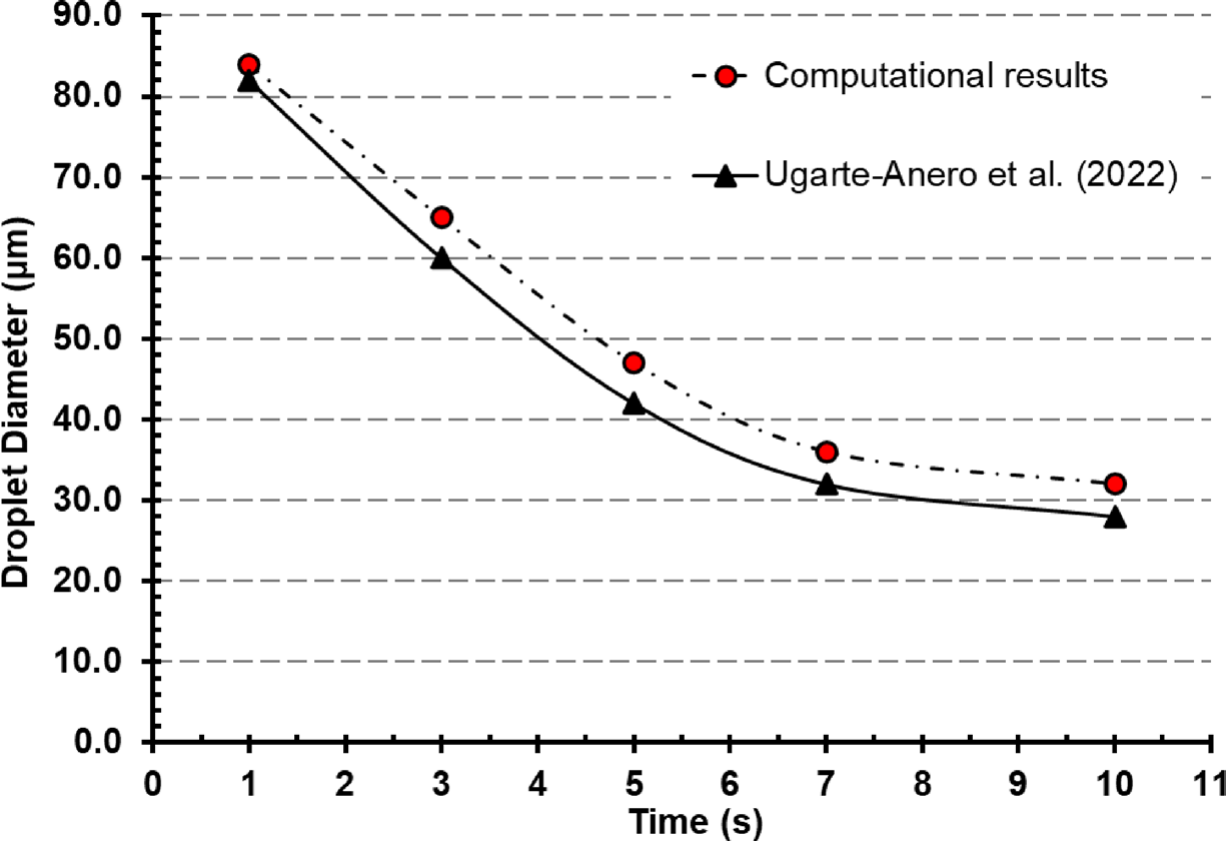
Validation of droplet diameter evolution over time. The simulation results from the current study are compared with data from Ugarte-Anero et al.^19^.

In addition to the conceptual validation based on the Wells evaporation–falling curve, which verifies the overall fate of droplets across different size ranges, a more detailed, time-resolved validation was performed by comparing our simulation with the study by Ugarte-Anero et al.^19^ (Fig. 11). In their CFD analysis, a saliva droplet with an initial diameter of 100 μm was tracked over a 10-s period in a mechanically ventilated indoor environment. Their findings showed a progressive reduction in droplet diameter to approximately 28 μm at 10 s, with key intermediate values of ~ 82 μm at 1 s, ~ 60 μm at 3 s, and ~ 42 μm at 5 s, reflecting the influence of evaporation and convective airflow. In our simulation, droplets of the same initial size followed a closely similar trend, decreasing to approximately 32 μm at 10 s. This close agreement between both datasets validates the evaporation kinetics and thermodynamic modeling implemented in the current study. Moreover, both simulations highlight the combined effects of droplet shrinkage, airflow entrainment, and gradual sedimentation, reinforcing the physical accuracy of our Lagrangian discrete phase model and the realism of the indoor environmental conditions simulated.

Although the present study employed a simulation time of 10 s, this duration was carefully selected to capture the initial dispersion phase of cough-generated aerosols—an interval that encompasses the most critical period for short-term exposure risk in mechanically ventilated environments. Previous studies, such as those by Li et al.^29^ and Zhao et al.^30^, have demonstrated that the dominant mechanisms influencing aerosol transport—including jet momentum, turbulent entrainment, evaporation, and near-field dispersion—occur within the first few seconds following a respiratory event. Therefore, the selected timeframe offers sufficient resolution to evaluate the immediate influence of ventilation strategies on aerosol behavior.

The accuracy of this approach has been validated against well-established experimental and theoretical benchmarks, including the Wells evaporation–falling curve^58^ , droplet diameter decay data from Ugarte-Anero et al.^19^, and cough cloud propagation characteristics described by Agrawal and Bhardwaj^57^. These comparisons confirm that the model reliably captures the key physical processes relevant to short-term aerosol dynamics.

While it is acknowledged that longer simulation durations and advanced modeling techniques such as Large Eddy Simulation (LES) can provide enhanced insight into long-term suspension, room-scale mixing, and re-entrainment phenomena, such approaches come with substantial computational demands. Given the need to compare multiple full-scale ventilation scenarios, the use of a validated Eulerian–Lagrangian framework coupled with RANS turbulence modeling was deemed appropriate. Future work may extend this modeling framework using LES and longer simulation windows to capture the broader temporal dynamics of aerosol behavior in more complex or naturally ventilated indoor environments.

## Numerical assessment and visualization

The simulation results presented in this section are based on a short-term analysis of aerosol dispersion following a cough event in a mechanically ventilated office environment. The numerical model employs a transient Eulerian–Lagrangian framework with a standard k-ε turbulence model to ensure computational feasibility across multiple ventilation scenarios. Several simplifications were applied, including the exclusion of thermal plumes, continuous breathing cycles, long-term aerosol suspension, and viral decay. Additionally, the 10-s simulation duration is intended to capture the initial dispersion dynamics, not the complete evolution of aerosol transport. These assumptions are suitable for evaluating ventilation-induced particle movement in high-flow conditions but may limit applicability to passive or thermally stratified environments. The findings should therefore be interpreted within the context of these constraints, with future studies recommended to incorporate LES models, thermal effects, and physiological boundary conditions for more comprehensive exposure assessment.

In comparison of the two cases simulated regarding the supply and exhaust diffusers placement as mentioned before, a more detailed analysis is presented in the upcoming section helping in understanding the cough dynamics and the air flow interaction to identify clearly the risk of exposure between the two cases of diffusers settings.

### Maximum travel distance

Evaluated by FLUENT assessing the track of particles inside the room to predict the maximum travel distance by droplets in the room. In.

**Fig. 12:**
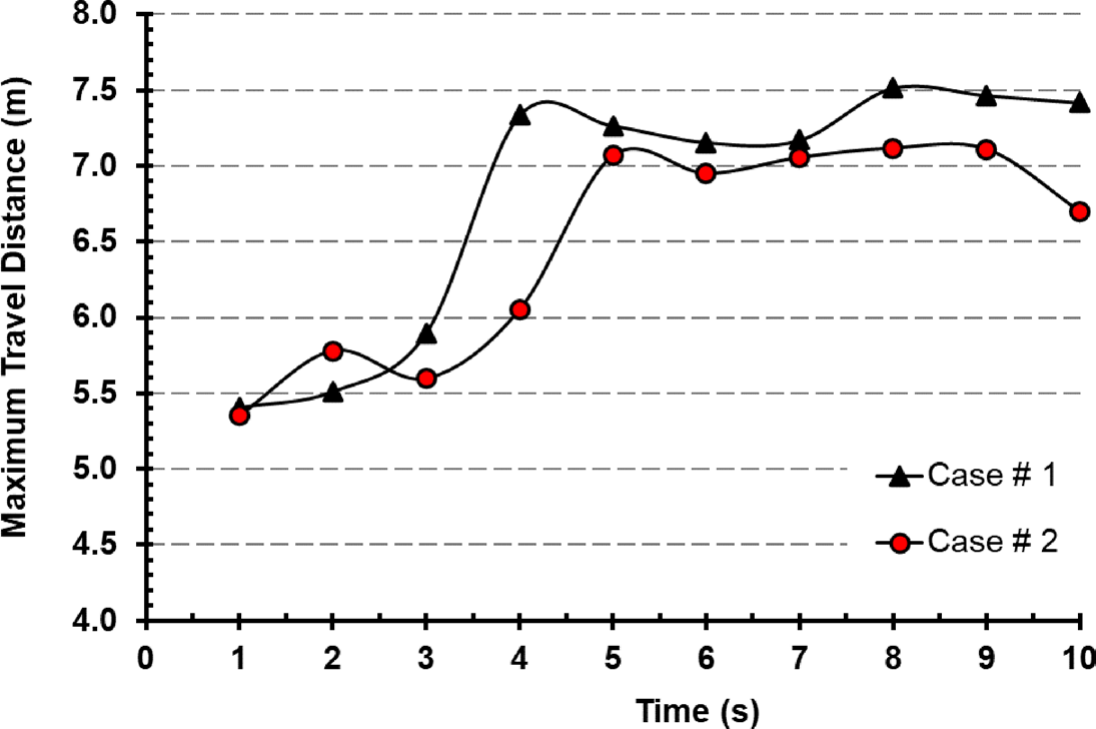
Maximum travel distance of cough droplets over time for Case # 1 and 2.

**Fig. 13:**
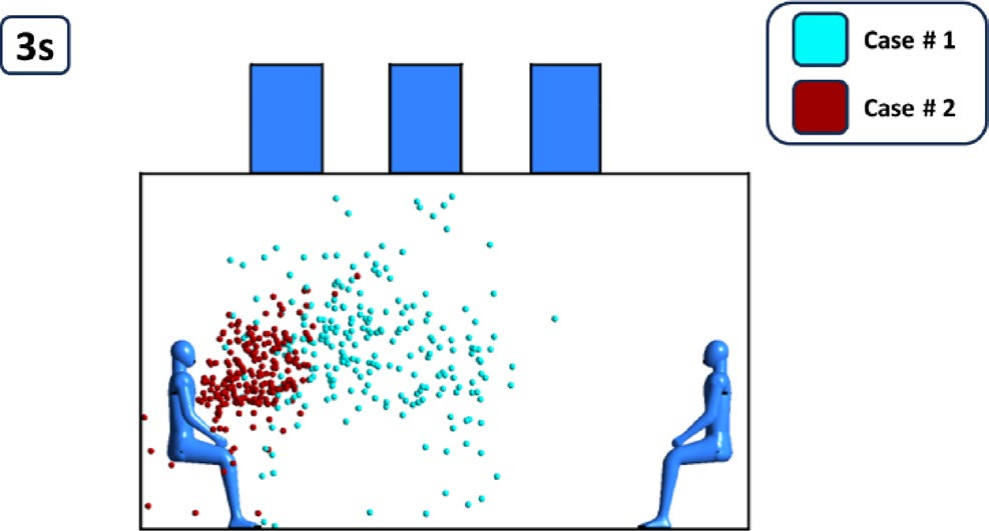
Particle tracking with the two cases in 3 s.

**Fig. 14:**
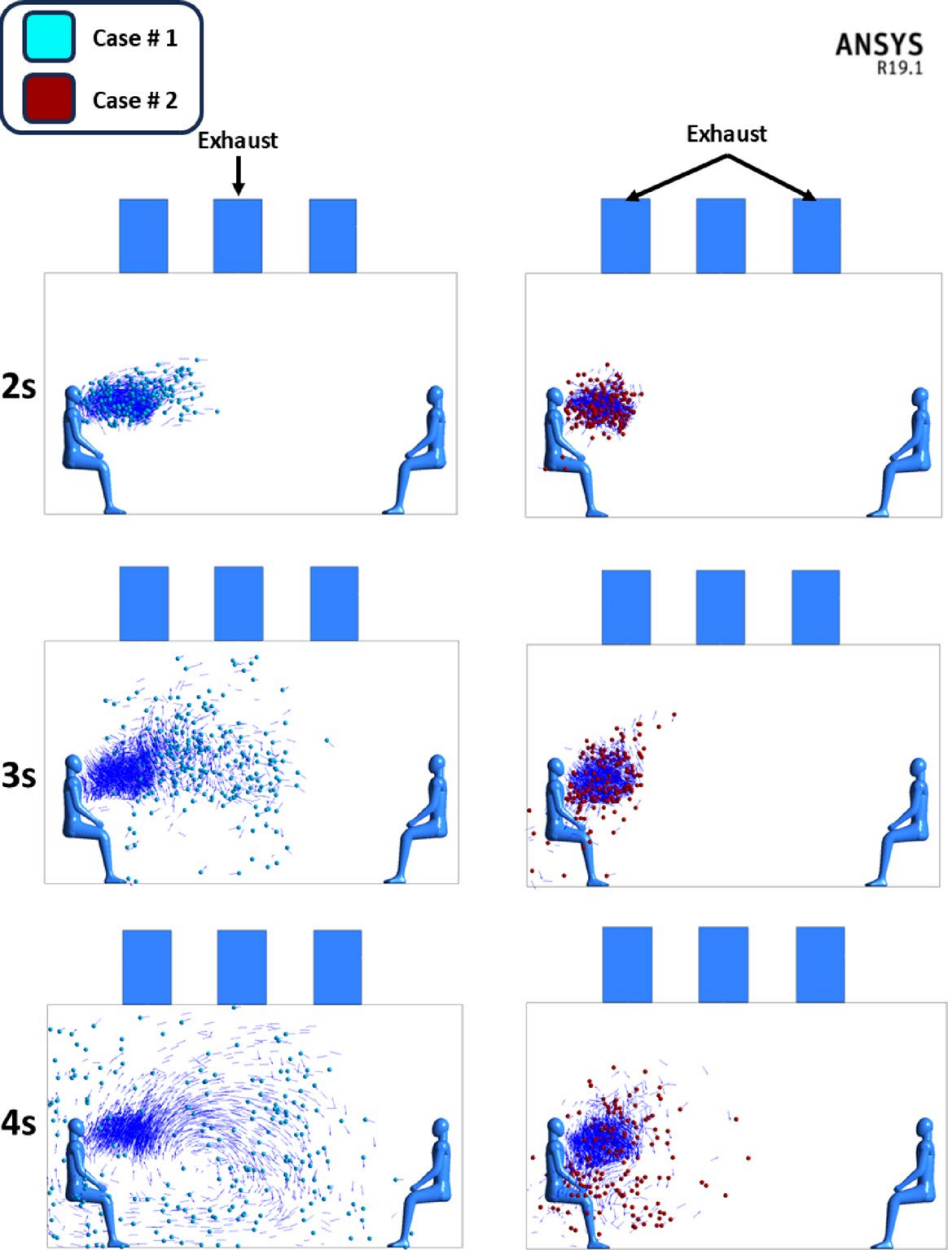
velocity vectors of cough particles in Case # 1 and case # 2 at 2, 3 and 4 s, created using ANSYS R19.1 (https://www.ansys.com).

Figure 12, the comparison between both cases had been presented to show the same trend and behavior for particles throughout the time frame simulated. On average, Case # 2 where the exhaust diffuser is above the cough source showed lower travel distance for particles. It had been noticed that in case # 2 the difference reached 10% on average when compared with case # 1. For further exploration and visualization for the previously mentioned figure a particle track shot were taken from FLUENT in the period of 3 s (Fig. 13), it is clear that in case # 2 the particle dispersion is lighter and inhibited with the supply diffuser above the cough source, while in Case # 1 particles is dragged more into the middle of the room towards the middle exhaust diffusers. With the aid of Fig. 14, showing the velocity vectors influence on cough particles on 2,3 and 3 s, it is evident that in Case #1, velocity vectors influence the particles towards the parallel diffusers in the middle of the particle cloud, growing it over a period of three seconds. However, in Case #2, most of the velocity vectors are directed upward (approximately 90 degrees) towards the exhaust diffuser on the left (Fig. 14), which prevents the particle cloud from extending and ensures the results seen in Fig. 12.

### Remaining droplet mass proportion

**Fig. 15:**
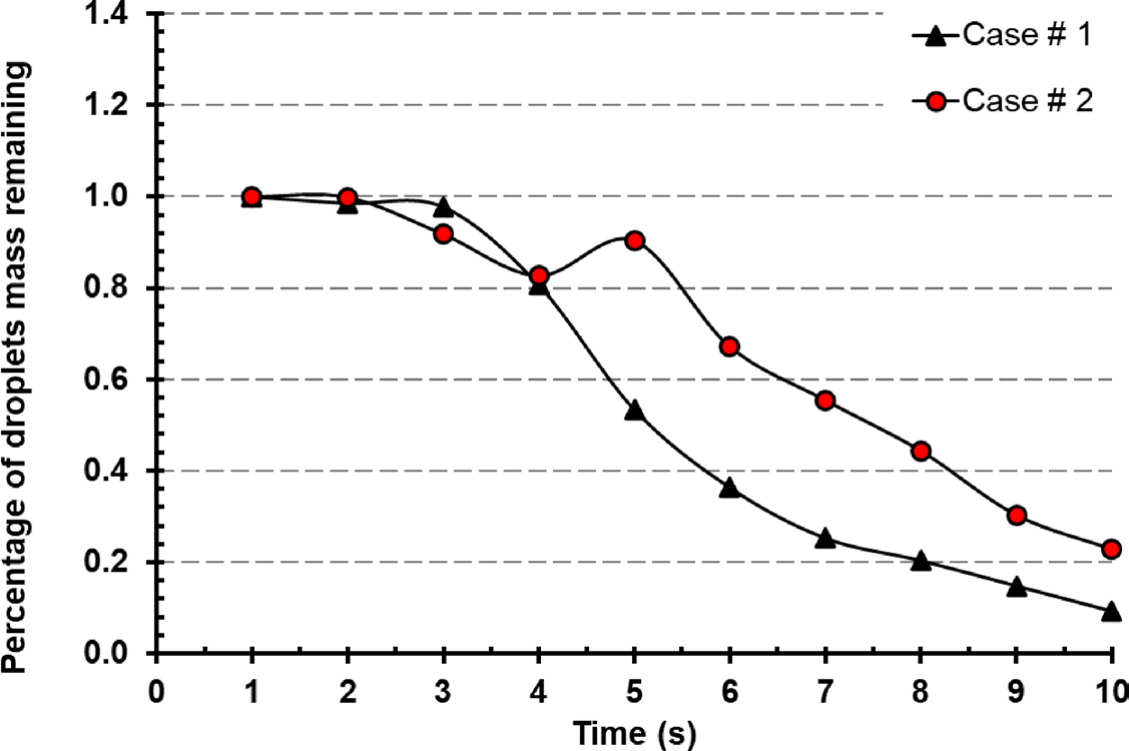
Droplets mass remaining percentage over time for Case # 1 and # 2.

Characterizing the viral concentration in the room may be done using the droplet mass that remains inside the room and had not been exhausted. It has been demonstrated by determining the relationship between inject droplet mass and residual droplet mass over time through displaying the proportion of droplets mass remaining in the two-diffuser configuration. In Fig. 15, both cases working perfectly in exhausting the droplets mass of the cough injected particles reaching 10% for case # 1 and 21% for case # 2 in 10 s simulation period. The power of parallel exhaust system for case # 1 configuration had been noticeable specially after 5 s period while the cough injection matured and traveled to nearly half of the room.

### Droplets Sauter mean diameter

**Fig. 16:**
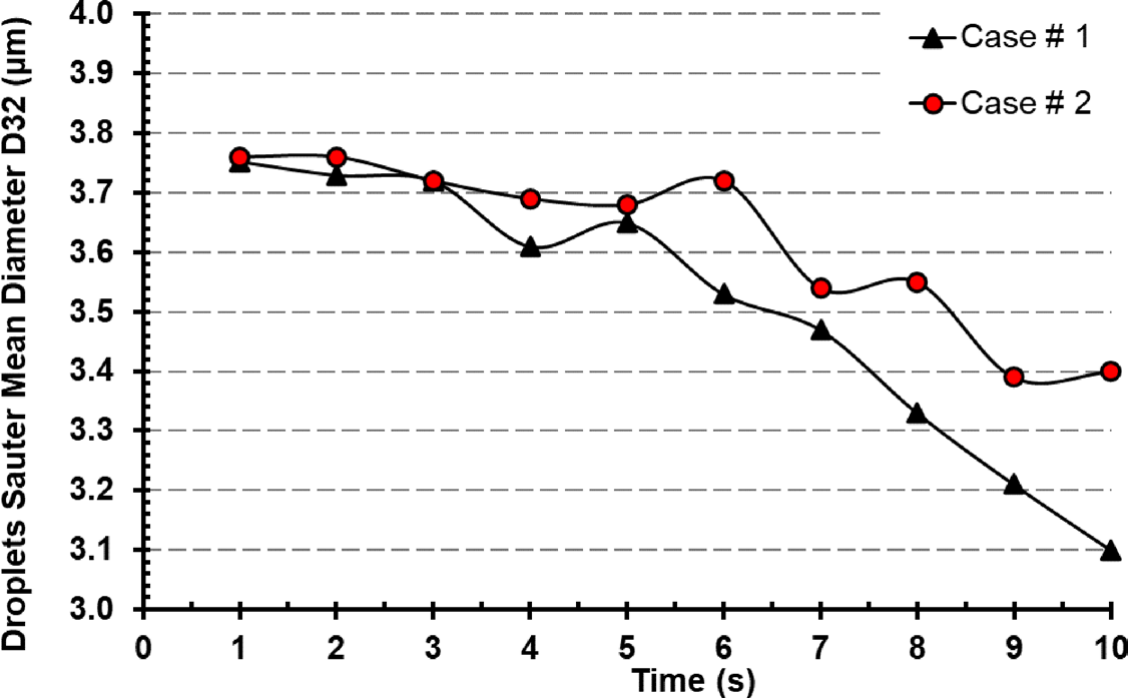
Droplets Sauter mean diameter over time for Case # 1 and # 2.

A more thorough quantitative analysis of the droplet lifetimes while diameter distribution of droplets in the room alters continuously because of the effects of droplet evaporation, sedimentation, and adhering on the different surfaces and walls. The transmission behavior of droplets is directly influenced by their diameter. To measure how droplet size changes over time, Sauter mean diameter (SMD), commonly referred to as D32, is used (Fig. 16). While analyzing Fig. 16, it has been found that the droplet diameter didn’t vary significantly when comparing case # 1 to case # 2 for the first 5 s. Although the variance is negligible case # 1 was superior in terms of the droplets Sauter mean diameter with a maximum of 2.7% difference at 4 s’s time. The difference was much noticeable in the last 5 s (5 < Time < 10) with an average value of 4% difference. The maximum difference occurred at the end of the period at 10 s when the difference reached 8.9%. Confirming the droplets mass remaining proportion findings for the power of the parallel exhaust system.

15 $$D_{32} = \frac{{\mathop \smallint \nolimits_{{D_{min} }}^{{D_{max} }} D^{3} dN}}{{\mathop \smallint \nolimits_{{D_{min} }}^{{D_{max} }} D^{2} dN}}$$

where *N* is the number of droplets having a diameter of *D*.

To assess the effectiveness of ventilation configurations and social distancing measures in reducing airborne transmission risks, a time-averaged particle concentration analysis was performed along the Z-axis planes at incremental distances from the cough source till it reaches the employees face at the end of the room as illustrated earlier in Fig. 1. This analysis aimed to identify the optimal diffuser arrangement for workplace environments and evaluate the impact of physical distancing on aerosol dispersion. The total number of particles, encompassing both droplets and aerosolized nuclei, at the cough source was used as the reference point, with measurements systematically taken at half-meter intervals throughout the simulation domain to provide a comprehensive evaluation of dispersion patterns.

**Fig. 17:**
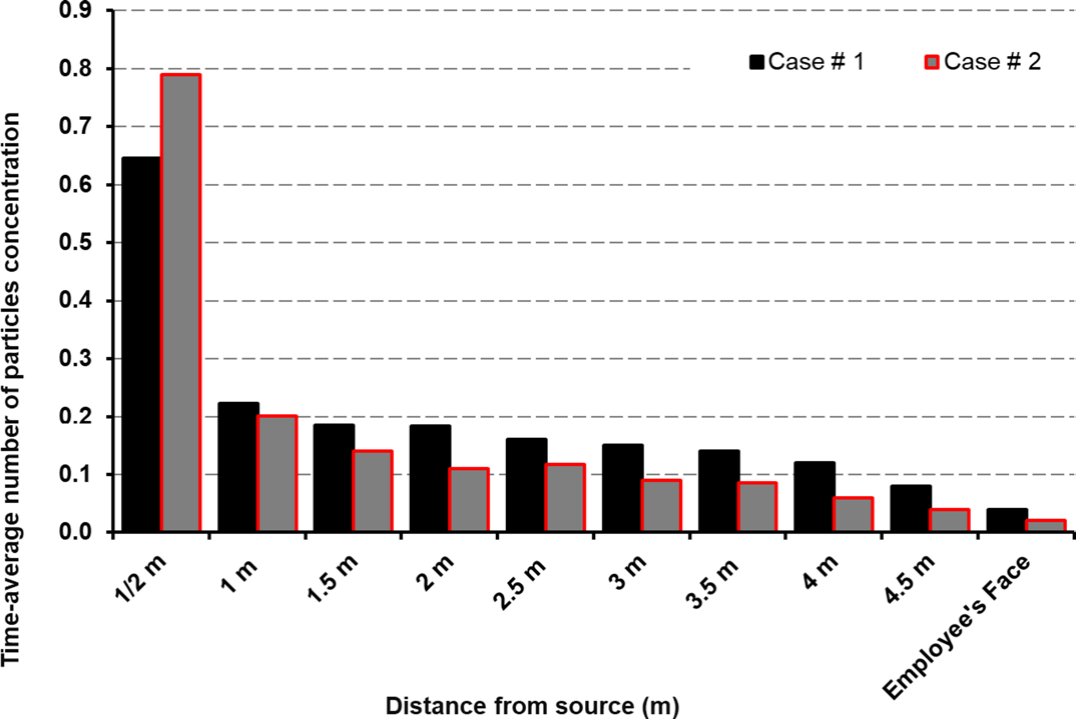
Time averaged number of particles concentration in planes from cough source till employee’s face.

A detailed examination of Fig. 17 reveals that at the 0.5-m plane, Case #2 exhibits a higher total particle concentration, encompassing both droplets and aerosolized nuclei, compared to Case #1. This observation aligns with the trends shown in Fig. 12, further confirming that positioning the exhaust diffuser directly above the cough source effectively reduces the lateral migration of expelled particles within the room. However, as the distance increases from 1 to 2.5 m, a notable decline in overall particle concentration is observed in both cases, highlighting the role of diffusion and dilution mechanisms in mitigating airborne particle dispersion. Observations beyond 2.5 m show a continued decline in airborne particle concentration, demonstrating the effectiveness of spatial distancing and airflow-driven dispersion. Between 3 and 4.5 m, the concentration progressively decreases, with Case #1 and Case #2 showing values of 0.12 and 0.11 at 3 m, respectively, and further reducing to 0.04 and 0.03 at 4.5 m, indicating that most expelled particles have either settled, diffused, or been removed through ventilation mechanisms. At the employee’s face, the concentration reaches its lowest values of 0.02 in Case #1 and 0.015 in Case #2, confirming a substantial reduction in inhalation risk at this position.

A comparative assessment reveals that Case #2 achieves a greater reduction in particle concentration, with a maximum drop of 40% relative to Case #1. However, as evidenced in Fig. 11, Case #1 excels in overall particle removal, demonstrating the parallel exhaust system’s efficiency in reducing the percentage of residual mass within the office space. Despite this advantage, the parallel exhaust configuration in Case #1 does not effectively limit lateral dispersion, allowing particles to travel further from the source.

A thorough analysis of Fig. 17, which specifically examines particle number concentration, underscores the significant reduction in airborne particle count with increased distancing in indoor office environments. In contrast, Fig. 11 focuses on mass concentration, providing insights into the effectiveness of ventilation strategies in removing airborne contaminants without taking into account the droplets nuclei. Deeper insight in simulation results indicate that maintaining a distance of approximately 2 m is associated with an 82% reduction in particle number concentration in Case #1 and an 89% reduction in Case #2. These findings align with established public health guidelines and prior studies^59–62^ , emphasizing that while spatial distancing plays a crucial role in mitigating airborne transmission risks, its effectiveness depends on multiple factors, including ventilation efficiency, airflow dynamics, and environmental conditions. This highlights the necessity of integrating optimized ventilation strategies alongside distancing measures to effectively minimize transmission risks in workplace settings.

### Study limitations and future directions

This study provides insights into the short-term dispersion of cough-generated aerosols in a mechanically ventilated office environment using CFD simulations. However, several methodological limitations must be acknowledged. First, the simulation duration was limited to 10 s, focusing on the early-phase dispersion dynamics immediately following a cough event. While this captures the critical window of peak exposure risk, it does not account for longer-term aerosol suspension, room-scale mixing, or viral decay processes. Second, the model employed a Reynolds-Averaged Navier–Stokes (RANS) framework with the standard k-ε turbulence model, which, while computationally efficient, may not resolve all transient flow structures as effectively as more advanced turbulence models such as Large Eddy Simulation (LES). Third, the study excluded the effects of body-generated thermal plumes, continuous inhalation–exhalation cycles, and particle re-entrainment, which could influence local airflow dynamics and exposure risk under certain conditions. Lastly, the simulation assumed a single emission source and did not incorporate biological decay or infection risk modeling. These limitations suggest that while the findings are valuable for understanding early-phase aerosol behavior and informing ventilation design strategies, additional research incorporating extended simulation times, refined turbulence models, and physiological factors is recommended to fully capture the complexities of airborne transmission in real-world settings.

## Conclusions

This study computationally analyzes airborne particle dispersion in offices, examining how ventilation configurations and spatial distancing mitigate respiratory virus transmission. Using CFD simulations, the study employs an Eulerian–Lagrangian approach with the Discrete Phase Model (DPM) to track cough-emitted droplets and nuclei, capturing their transport, evaporation, and interaction with airflow.

Key findings indicate that:

- Exhaust placement above the cough source effectively reduces lateral migration of expelled particles, preventing their spread across the room.
- The parallel exhaust configuration enhances overall particle removal but does not effectively limit lateral dispersion, allowing particles to travel further.
- Maintaining a 2-m social distancing measure proves to be an effective strategy, as our results demonstrate an 82% reduction in particle number concentration in Case #1 and an 89% reduction in Case #2 at this distance. This confirms that 2 m serves as a significant threshold for reducing airborne exposure risk, particularly when combined with proper ventilation.
- Extending the analysis to 4.5 m, including the employee’s face, further confirmed a continued decline in particle concentration, reinforcing the importance of integrating ventilation optimization with spatial distancing measures.

By employing the Eulerian–Lagrangian method, this study effectively simulates interaction between airflow and discrete-phase particles, while the DPM model accurately tracks the motion, deposition, and evaporation of cough-generated droplets. This methodology enables a detailed assessment of airborne pathogen transport under varying ventilation strategies. Our findings underscore that 2 m is a reasonable and effective social distancing measure for minimizing airborne transmission risks, particularly when implemented alongside well-designed ventilation strategies. These insights offer concrete, engineering-driven guidance for public health interventions in office environments. By bridging the gap between advanced fluid dynamics simulations and practical applications in building environmental design and occupational health, this work contributes to the development of more effective indoor air management protocols, ultimately enhancing workplace safety and resilience against future respiratory pathogen outbreaks.

Importantly, this study is focused on the early-phase dispersion of aerosols following a cough event, a critical period for understanding immediate exposure risks and informing effective ventilation strategies. While long-term aerosol suspension, viral decay, and infection risk modeling are beyond the scope of this work, our results provide valuable insights into the design of HVAC systems and office layouts to reduce short-term transmission risk. This research lays a solid foundation for future studies that can build upon these findings by incorporating extended simulation times, advanced turbulence models, and biological considerations to comprehensively assess airborne transmission dynamics in indoor environments.

## Data Availability

The research data supporting this study is available upon request. For access, please contact Mina Saad at mina.saad@aast.edu.

## Abbreviations

CFD: Computational fluid dynamics
COVID-19: Coronavirus disease 2019
HVAC: Heating, ventilation, and air conditioning
MERS-CoV: Middle-east respiratory syndrome coronavirus
SARS-CoV: Severe acute respiratory syndrome coronavirus
SARS-CoV-2: Severe acute respiratory syndrome coronavirus 2
UV-C: Ultraviolet-C
RH: Relative humidity
SMD: Sauter mean diameter
°C: Degrees celsius
D: Diameter
M: Meters
*N*: Number of droplets
s: Seconds

## List of symobls

*μ*: Micro
X: Length
Y: Width
Z: Height
$k$: Turbulent kinetic energy
$\varepsilon$: Rate of dissipation of turbulent kinetic energy
$$u$$: Velocity
${\tau }_{r}$: Droplet relaxation factor
$$\overrightarrow{g}$$: Gravitational acceleration
$$\rho$$: Droplet density
$\zeta$: Normally distributed random number
